# Cross cultural verbal cues to deception: truth and lies in first and second language forensic interview contexts

**DOI:** 10.3389/fpsyg.2023.1152904

**Published:** 2023-05-30

**Authors:** Coral J. Dando, Paul J. Taylor, Alexandra L. Sandham

**Affiliations:** ^1^Department of Psychology, University of Westminster, London, United Kingdom; ^2^Department of Psychology, Lancaster University, Lancaster, United Kingdom; ^3^Faculty of Applied Social Sciences, Open University, Milton Keynes, United Kingdom

**Keywords:** detecting deception, plausibility, first and second language, cross cultural, South Asian

## Abstract

**Introduction:**

The verbal deception literature is largely based upon North American and Western European monolingual English speaker interactions. This paper extends this literature by comparing the verbal behaviors of 88 south Asian bilinguals, conversing in either first (Hindi) or second (English) languages, and 48 British monolinguals conversing in English.

**Methods:**

All participated in a live event following which they were interviewed having been incentivized to be either deceptive or truthful. Event details, complications, verifiable sources, and plausibility ratings were analyzed as a function of veracity, language and culture.

**Results:**

Main effects revealed cross cultural similarities in both first and second language interviews whereby all liar’s verbal responses were impoverished and rated as less plausible than truthtellers. However, a series of cross-cultural interactions emerged whereby bi-lingual South Asian truthtellers and liars interviewed in first and second languages exhibited varying patterns of verbal behaviors, differences that have the potential to trigger erroneous assessments in practice.

**Discussion:**

Despite limitations, including concerns centered on the reductionary nature of deception research, our results highlight that while cultural context is important, impoverished, simple verbal accounts should trigger a ‘red flag’ for further attention irrespective of culture or interview language, since the cognitive load typically associated with formulating a deceptive account apparently emerges in a broadly similar manner.

## 1. Introduction

The forensic interviewing literature concerned with distinguishing liars from truth-tellers is largely based upon North American and Western European research and has typically focused on monolingual English speaker interactions (see Granhag and Strömwall, 2004; Castillo and Mallard, 2012; Laing, 2015; Leal et al., 2018). Yet, the transnational nature of criminal investigation is such that forensic interviewers regularly encounter persons of interest from diverse cultures. Culture can be defined as ‘the collective programming’ that distinguishes one group from another (Hofstede and Hofstede, 2005) and so culture typically refers to characteristic societal markers that determine individual attitudes, behaviors and values (Lytle et al., 1995). Accordingly, individuals from different cultures communicate variously, often differing in factors such as their degrees of verbal directness, cohesion and coherence, pacing and pauses, and what to say (e.g., Levine et al., 2007; Reynolds et al., 2011; Taylor et al., 2014).

Psychological understanding of verbal deception in these ‘cross-cultural’ interactions is not well advanced, despite verbal behavior being known to be culturally diverse. Hence, cultural variability adds further to the well documented challenges of detecting verbal truth and lies in forensic interview contexts, which in part emanates from the inconsistent nature of verbal cues in general (see Porter et al., 2000; Dando and Bull, 2011; Vrij, 2014; Sandham et al., 2020). Even when interviewer and interviewee share the same first language, differences in expected norms and cultural speech practices are sufficient to trigger misunderstandings, both in face-to-face dialogue (van der Zee et al., 2014; Giebels et al., 2017; Taylor et al., 2017) and in computer mediated interactions (e.g., Levine et al., 2007; Durant and Shepherd, 2013; Hurn and Tomalin, 2013).

Additional challenges can arise when one or both interviewers are required to speak in a second language, since this complicating verbal communication still further (see Cheng and Broadhurst, 2005; Da Silva and Leach, 2013; Duñabeitia and Costa, 2015). While English is the most widely spoken language worldwide, for many, it is spoken as a second language and so it is not uncommon for cross cultural interviews to be conducted in English. Irrespective of veracity, the additional cognitive demands associated with communicating in a second language (Segalowitz, 2010; Tavakoli and Wright, 2020) may trigger verbal cues that research on first language interviews identifies as diagnostic of truth and lies. For example, research has indicated that liars often provide impoverished, simple verbal accounts, which lack verifiable information and contain proportionally fewer event details and complications than truth-tellers (e.g., Leal et al., 2018; Vrij et al., 2020; Vrij and Vrij, 2020). Although some of these verbal cues appear stable across cultures (Vrij and Vrij, 2020), as far as we are aware the forensic verbal deception literature has yet to fully investigate first and second language variances.

### 1.1. Deceptive and truthful verbal communication

Cognitive theories of deception predict differences in verbal behaviors because lying can be more difficult than being truthful. For example, Cognitive Load/Effort theory (Vrij, 2000) posits that lying is often a more complex mental activity than telling the truth since liars must manage the numerous concurrent cognitive demands associated with (among other things) withholding the truth, formulating a deceive account, matching accounts to known or discoverable information, appearing plausible, and maintaining consistency (e.g., Buller and Burgoon, 1996; Hartwig et al., 2005; Vrij et al., 2011; Leins et al., 2012; Vrij et al., 2012; Verigin et al., 2020). Consequently, liars may offer impoverished and vacuous accounts in response to questions versus truthtellers as a way of managing their deception.

Indeed, the amount of event information provided in verbal accounts has consistently emerged as a useful cue to veracity, with truthtellers typically providing more detail in their accounts than deceivers (e.g., DePaulo et al., 2003; Vrij, 2008; Taylor et al., 2013; Dando and Ormerod, 2020; Dando C. J. et al., 2022). Operationalizing this cue can be challenging, since researchers typically use interview transcripts, rather than having to be alert to deception in real time. Although interviewers are not generally required to make veracity decisions, they are expected to be alert to deception and so concerns about the cue’s utility are valid. Nonetheless, the amount of event detail provided has been found to cue more accurate truth and lie decisions in both face-to-face and remote interview contexts (e.g., DePaulo et al., 2003; Vrij, 2008). Further, researchers have reported that both professionals and some lay persons are able to recognize information poor and impoverished accounts, and so are socially alert to this verbal behavior (e.g., Vrij and Mann, 2004; Verigin et al., 2020).

The amount of verifiable source information provided, the complexity of answers, and the overarching plausibility of the account are related factors that have also emerged as veracity cues (see Vrij and Vrij, 2020; Vrij et al., 2022). Verifiable sources, or ‘concrete’ details that *could* be verified from witness statements, CCTV, and trace evidence for example (see also Ormerod and Dando, 2015), are often more common in truthful accounts (e.g., Vrij et al., 2020; Leal et al., 2022). Complications are details provided that serve to complicate an account by adding uninvited additional event relevant detail. For example, *‘I went up to the food counter, which had a basket of fruit on top. The fruit looked really lovely. I remember there were bananas, which I really love’* rather than *‘I went to the food counter’*. Again, research has indicated that truthful accounts often include more complications (e.g., Vrij et al., 2021b) suggesting paucity of complications is indictive of deception.

Plausibility, in terms of judging how ‘likely’ or ‘believable’ an account is (e.g., DePaulo et al., 2003; Leal et al., 2019), is a subjective assessment/rating. Nonetheless, plausibility judgments have been found to distinguish truth tellers from liars whereby plausibility ratings of deceptive accounts are typically significantly lower (e.g., DePaulo et al., 2003; Vrij et al., 2021a). Furthermore, plausibility ratings using a Likert scale were found to positively predict details, complications and verifiable sources indicating observers recognized these verbal behaviors differed (Vrij et al., 2021a). Indeed, many interview techniques developed toward amplifying cues to detection in real time have drawn on the notion that truthful accounts *should* be more plausible and make more sense and so have focused on credibility cues (e.g., Dando and Bull, 2011; Granhag and Hartwig, 2014; Ormerod and Dando, 2015). It appears professional observers and interviewers are able to recognize improbable accounts in some circumstances, particularly when interviewers employ techniques to amplify credibility cues (e.g., Dando et al., 2009, 2018; Vrij et al., 2009; Evans et al., 2013; Lancaster et al., 2013).

Our understanding of how consistency, verifiability, plausibility and complications relate to veracity is more advanced for North American and Western European participants than for other populations. It seems sensible to assume, however, that theories of Cognitive Load/Effort are relevant irrespective of culture, since cognitive processes such as memory and attention are universal. What is less clear is how cognitive load will manifest for different cultures. For example, Taylor et al. (2017) found that liars with North African cultural backgrounds tended to increase their provision of perceptual details when lying, with this supplanting their cultural norm of providing social details. The opposite was true for liars from Western Europe. Conversely, some researchers have reported more event details and checkable sources are provided by truthtellers irrespective of language (see Ewens et al., 2016, 2017; Leal et al., 2018). For example, Russian, Korean and Hispanic truthtellers were found to include more complications than liars when providing travel accounts (Vrij and Vrij, 2020). These differing findings illustrate how nascent this area is, with a paucity of interview relevant research findings.

### 1.2. Truth and lies in first and second languages

Those few studies of veracity across second language and bilingual communication suggest that expectations, the cues attended to, and language (first versus second) all impact veracity judgment performance. Bilinguals experience heightened cognitive load when being both deceptive and truthful in a second language (Da Silva and Leach, 2013; Akehurst et al., 2018) and so verbal cues to veracity such as low information, reduced complexity, and fewer verifiable sources may be apparent but not necessarily associated with lying. However, laypersons and professionals (police officers) appear to believe liars communicating in both first and second languages are likely to exhibit the similar verbal veracity cues (Leach et al., 2020). They also expect differences in interview length due, for example, to misunderstanding of questions and delayed response times (Leach et al., 2020); this has been borne out by increased response durations when being deceptive in a second language versus first language (McDonald et al., 2020).

Despite expectations of similar verbal behaviors, a lie bias has begun to emerge when judging non-native (second language) speakers. In contrast, a truth bias is more evident when judging native (first language) speakers (Da Silva and Leach, 2013; Evans and Michael, 2014; Wylie et al., 2022). Similarly, veracity judgment accuracy is better when judging first vs. second language speakers (Da Silva and Leach, 2013; Taylor et al., 2014; Leach et al., 2017; Akehurst et al., 2018), although not always. Others have reported improved veracity judgments in second language contexts (e.g., Evans et al., 2013), or no discernable differences (Cheng and Broadhurst, 2005) as a function of language (First Cantonese; second English), although in this research the language status of the observers is not always clear.

### 1.3. The current research

The research reported here seeks to advance our understanding of the occurrence and potential cueing utility of details, verifiable details, complications, and plausibility as verbal veracity cues in forensic interview contexts, with bilinguals from a non-western culture. Specifically, monolingual (British) and bilingual (South Asian) participants took part in a laboratory task that involved carrying out an activity (that differed in part as a function of liar or truthteller condition), following which they were interviewed in either their first (English and Hindi) or second (English) language. All deceptive participants self-generated an account to convince the interviewer that they had completed the same activity as the truthful participants. Interviewers and interviewees were culture and language matched. Interview transcripts were coded and rated for plausibility.

The relevant literature is sparce and the findings are mixed. Hence, we formulated a series of questions driven not only by a clear need to advance understanding of verbal behaviors across different cultures with reference to the real-world challenges and associated empirical questions raised by professionals/practitioners tasked with maximizing opportunities to better understand truth and lies. It is these research questions and challenges that guided both our paradigm and analysis approach, as follows.

First, using first language (L1) as a proxy for operationalizing culture, we examined the occurrence of verbal cues (event details, complications, and verifiable sources) and plausibility as a function of veracity. Consistent with previous research, we expected truthtellers to present more of each cue than liars irrespective of cultural background.

Second, we examined the occurrence of verbal cues and plausibility when interviewed in a first versus a second language as a function of veracity. Consistent with previous research, we expected the behavior of second language speakers to include less of the verbal cues than the first language speakers.

Third, we examined cultural differences and similarities in the occurrence of verbal cues and plausibility across cultures as a function of veracity. While we recognize the inconsistencies of prior research in this area, we expected that judgments of plausibility would be particularly impacted for the those interviewed in their second language since empirical evidence has begun to emerge of lie bias for second language speakers (see Wylie et al., 2022).

## 2. Materials and methods

### 2.1. Participants

An *a-priori* power analysis was conducted using G*Power 3.1 (Faul et al., 2007) to determine minimum sample size estimation. Power analysis for ANOVA: main effects and interactions for three groups with a numerator df of 2 indicated the required sample size of mock witnesses to detect large effects (assuming power = 0.80 and *a* = 0.05) was *N* = 121. Thus, the obtained sample size of *N* = 136 was adequate given resource constraints and access to bilingual populations and is in line with sample size norms described in many empirical cross cultural studies such as the one reported here (e.g., Al-Simadi, 2000; Cheng and Broadhurst, 2005; Castillo and Mallard, 2012; Evans et al., 2017; Primbs et al., 2022). Participant interviewees were recruited through word of mouth, social media and advertisements placed in the locality of the University. This research was approved by Lancaster University’s Psychology Ethics Committee and was run in accordance with the British Psychological Society code of ethical conduct.

#### 2.1.1. Interviewees

A total of 136 adults took part in this research (64 males and 72 females). The Mean age was 22.13 (*SD* = 2.14), ranging from 18 to 29 years. There were 88 (64.7%) bi-lingual participants with Hindi as their first language and English as a second language (41 male and 47 female) and 48 (35.3%) monolingual English speakers (23 male and 25 female). Participants were randomly allocated to either the liar or truthteller veracity condition, resulting in 70 liars (51.5%) and 66 Truthtellers (48.5%). Bilingual participants were further allocated to one of two interview language groups, namely first language (Hindi) or second language (English). Accordingly, there were six conditions (i) Monolingual British liars (25 participants), (ii) Monolingual British truthtellers (23 participants), (iii) Bilingual first language interview liar (22 participants), (iv) Bilingual first language interview truth (23 participants), (v) Bilingual second language interview truthtellers (20 participants), and (vi) Bilingual second language interview liar (23 participants). There were no significant differences in age across the groups, *F*(5, 130) = 0.621, *p* = 0.684, nor differences in gender distribution, *X*^2^ (5, *N* = 136) = 1.450, *p* = 0.919.

#### 2.1.2. Interviewers

Two female volunteer research assistant interviewers (from here on referred to as interviewers) took part in the research as interviewers (aged 22 and 24 years), one bilingual (Hindi and English Language) and one monolingual female (English language). The monolingual interviewer, a British citizen, born in the UK, employed at an English University, conducted all monolingual English interviews. The bilingual interviewer, a second generation British Indian, conducted all interviews with bilingual participants according to language condition. Both interviewers underwent bespoke training over a 2-day period. Training was designed for this research by the first author, adopting a collaborative pedagogical approach, comprising: (i) a 2-h long classroom-based introduction to the interview protocols behaviors, (ii) a 2-h long practice session that included 3 practice interviews, which were digitally recorded to allow feedback and evaluation. Once the interviewers had attended the classroom training sessions (training day 1) and completed the practice interviews to required level of competency, (training day 2) they were able to commence research interviews. Interviewers were naïve to the veracity conditions and hypotheses.

### 2.2. Materials

#### 2.2.1. Language and background questionnaire

Prior to participation all participants completed a 10-item hard copy self-report language proficiency and background questionnaire to guide groupings of 1st and 2nd language conditions (Supplementary materials OSF). Monolingual participants were all British citizens, born in the UK, with English as their first/only language. Bi-lingual participants were all Indian citizens born in India, attending a UK university to study at PG level. None of the bilingual participants (*n* = 88) self-reported having spent any time learning or working in another English-speaking country before the age of 16 years and reported starting to learn English at a mean age of 9.51 years (*SD* = 2.16, ranging from 6 to 15 years). On a Likert scale ranging from 1 (extremely poorly/never) to 7 (extremely well/always), bi-lingual participants reported that they spoke English well (*M* = 5.64, *SD* = 0.93), always spoke Hindi at home as a child (*M* = 7.00, *SD* = 0.00), always spoke Hindi with their parents (*M* = 7.00, *SD* = 0.00), always spoke Hindi at school (*M* = 6.78, *SD* = 0.53), and always spoke Hindi with friends (*M* = 6.79, *SD* = 0.55). The mean number of years spent completing formal education in English was 3.84 (*SD* = 1.19). All bilingual participants reported the language spoken at their first place of education was Hindi. Bilingual participants (Supplementary materials OSF) were asked which language they preferred to use (Hindi, English, or either/both) in various contexts while studying and living in England (see Table 1).

**Table 1:** Bilingual participant language preferences.

	English	No preference	Hindi
Home	4 (2.9%)	40 (29.4%)	44 (32.4%)
Work	7 (5.1%)	61 (44.9%)	20 (14.7%)
University	53 (39%)	31 (22%)	4 (2.9%)
Friends	43 (31.6%)	37 (27.2%)	11 (8.1%)
On Line	10 (7.4%)	67 (49.3%)	11 (8.1%)

#### 2.2.2. Post interview questionnaire

Immediately post interview, participants completed a hard copy questionnaire comprising a series of Likert scale questions ranging from 1 (very little/extremely easy/not at all) to 7 (very much/extremely hard/extremely motivated). Questions concerned adherence to the pre-interview instructions, motivation, experienced difficulty, and understanding.

#### 2.2.3. Interview protocol

Irrespective of condition, all interviews were similarly structured and comprised three information gathering phases, in the same order. First, participants were asked to provide a free recall account of everything they could remember, followed by a series of probing questions, finishing with a second free recall account (see Table 2). Explain and rapport building phases preceded the formal information gathering phases. Interviews finished with a closure phase (see Table 2).

**Table 2:** Interview phase description.

Phase	Overview
1. Explain	Introductions, explain the interview process and procedure and offer participants the opportunity to ask questions.
2. Rapport	Interviewer verbally interacts with the participant using two types of behaviors: Open-ended invitations to exchange information. For example, ‘*Thank you for coming to the University today. Do you work here, or do you study here?’* Offering some non-personal information to begin this process. For example, *‘Oh ok, do you like your course. I have many friends on the same course, actually. You might know them. They love it;’* Interviewer displays one attentive physical behavior: Nodding when interviewees speak/answer questions. Interviewer displays one attentive verbal behavior: Thanking interviewees whenever they provided information and answered a question. For example, *‘Thank you, that was useful in helping me to understand’* The attentive verbal and physical behaviors continued throughout the interview
3. Free recall	Commenced with an explanation of the four ground rules: Report all/everything Do not guess Say if you do not know Say if you do not understand Participants were then instructed to explain everything about their involvement in the events leading up to and after the theft of £5.00 from the researcher’s wallet. Once interviewees had finished, all were asked if they wished to add anything else.
4. Questions	Commenced with a reminder of the four ground rules (above), following which participants were asked four Tell Explain Describe (TED) prefaced probing question: Tell me about the conversation you had with the person you met in the University bar. Describe the University bar to me. Explain the route you took from the room where you met the research assistant to the University bar. Describe what you could see out of the window nearest to where you were sitting in the University bar.
5. Free recall	Commenced with a reminder of the four ground rules (above) following which interviewees were again instructed to explain everything about their involvement in the events leading up to and after the theft of £5.00 from the researcher’s wallet. Once interviewees had finished, all were asked if they wished to add anything else.
6. Close	Participants were thanked and offered the opportunity to ask questions.

### 2.3. Procedure

Participants were recruited to take part in an unspecified activity and then to take part in an interview following the activity. They were warned that they may be asked to deceive the interviewer as part of the interview but were naïve to the real aims of the project. All participants were asked to meet Researcher A (a confederate) in a café on the ground floor of a university building. Researcher A instructed the participant to deliver a package to Researcher B (also a confederate) who would to be waiting in an office on the third floor of the building. The package was marked confidential. It was explained to the participant that the package contained some important documents. Hence, once the package had been delivered to Researcher B it was vital the participant return to Researcher A, who would be waiting outside of the café in the courtyard, with proof of safe delivery in the form of a signed receipt. Researcher A then told participants that some money had gone missing and that they were going to be interviewed about it. Each participant was given 10 min to prepare for the interview.

Participants in the truth condition arrived at the 3rd floor office and were met by Researcher C (a confederate) who explained that Researcher B was running 15 min late and so could not sign for the package just yet. However, Researcher C suggested they go downstairs to the café until the researcher returned. Participants in this condition accompanied Researcher C to the café, where they had a coffee (or similar) and chatted about a series of general topics (e.g., University, where they lived, whether they had visited nearby cities etc.). After approximately 15 min Researcher C and the participant returned to the 3rd floor office. Researcher B was waiting and took the package from the participants and provided a signed receipt, which the participants took back to researcher A (back downstairs in the café), as instructed.

Participants in the deception condition however, upon arrival at the 3rd floor office, were immediately told by another confederate that the intended recipient (Researcher B) had just left the office but that they should not wait for his return, because it would seriously delay delivery of the package. Instead, they were instructed to deliver the package themselves to a courier who was waiting outside the building, but before doing so to forge Researcher B’s signature on the proof of delivery receipt which should then be returned to Researcher A as directed. The participant was instructed to forge the signature by copying Researcher B’s signature from his bank card that was in his wallet on the office desk. They were further instructed to take £5 from Researcher B’s wallet to give to the courier. Deceptive participants all completed this task as instructed. Once the Deceptive participants gave the signed receipt to Researcher A they were told that some money had gone missing and that they were going to be interviewed about it. Researcher A gave the Deceptive participants 10 min to develop a “plausible” explanation of them being in the café with Researcher C for a coffee and were told that their role was to persuade the interviewer that they were being truthful.

Each participant was then interviewed about the theft of £5 from Researcher B’s office. Two interviewers (one monolingual and one bilingual) conducted all interviews. Monolingual participants were all interviewed in English by the same western monolingual interviewer. The bilingual interviewer conducted all bilingual interviews in participant’s first (Hindi) or second language (English), randomly allocated across veracity conditions (lie and truth).

### 2.4. Coding

#### 2.4.1. Interview coding

Interviews were digitally audio recorded. English interviews were transcribed verbatim. Hindi interviews were first translated, and then transcribed. Transcriptions were coded for event details, verifiable information, and complications by a group of 10 British monolingual coders (each coding between 12 and 15 transcripts), all of whom were naïve to the experimental conditions and hypotheses. Coders comprised a group of post graduate research students, with experience of coding transcripts for information items with reference to a set of coding instructions. Prior to coding, all coders took part in two classroom-based group training sessions (each coder attending both sessions) lasting 2 h per session. Coding training was run by the first author and comprised (i) instruction/teaching on coding in general, (ii) project specific coding instructions, (iii) group coding of sample transcripts with feedback, (iv) individual coding of transcripts with feedback and group discussion regarding agreement and managing disagreement across coders, and (v) plausibility coding explanation/instruction. Coders also rated each transcript for plausibility. Items in each of the categories were only scored once (i.e., repetitions were not scored). Each of the 10 coders had therefore independently coded a minimum of three of the same transcripts.

Guided by the approach to coding employed by Leal et al. (2018) and Vrij and Vrij (2020), we counted the number of verifiable sources provided. Verifiable source information concerns verbalizations that could be used to verify the information provided by interviewees during the interview, such as named individuals, CCTV footage, text and phone conversations, purchasing information. For example, ‘*I went to the lab on the second floor, scanned in using my student ID and then logged onto my emails’* includes 2 verifiable sources (underlined) that could be accessed to verify what the participant said. Event information details were defined as a unit of detail/information about the café paradigm event (from start to end) and included all visual, spatial, temporal, auditory, and action details. For example, *‘There was a desk and three chairs. There was a middle-aged* *man* *sitting on the middle chair. He was talking to someone on the phone. We spent* *20 min* *in the café. XXX brought me a coffee, and packet of crisps. After a while, XXX got a call telling us to go back upstairs’,* includes 17 event information items. We defined a complication as a verbalization that serves to make the account of the event more complex and detailed. For example, ‘*I was talking to XXX when I asked if we could move because of the fridge in the corner. The light inside was so bright I almost wanted to put sunglasses on!’,* includes two complications. Information items, verifiable sources and complications were only coded once in that each was assigned to one of the verbal cues categories, only. Repetitions within each category were not coded. Plausibility (see Vrij et al., 2020) was rated using a 7-point Likert scale, asking coders to rate how ‘believable/plausible’ the account was (1 = not at all believable/plausible; 7 = completely unbelievable/unplausible).

Thirty of these transcripts were randomly selected. Two-way mixed effects Intraclass Correlation Coefficient (ICC) for agreement between multiple (10) research raters for event details, verifiable sources and complications were conducted. Mean estimates with 95% CI revealed very good inter-rater reliability for (i) event details, ICC = 0.994 (95% CI 0.991; 0.997), (ii) verifiable sources, ICC = 0.894 (95% CI 0.836; 0.940), and (iii) complications, ICC = 0.920 (95% CI 0.876; 0.955).

#### 2.4.2. Adherence to interview protocol coding

The same sample of 30 interviews were coded by an additional two independent coders for interviewer adherence to the interview protocol using a scoring sheet, which listed each of the required interviewer behaviors (i) inclusion of the 6 phases in the correct order, (ii) explaining the ground rules correctly, (iii) implement the four ground rules at the start of all three information gathering phases, (iv) asking four TED questions, and (v) using verbal rapport building behaviors in the rapport phase. Behaviors were coded, ranging from 1 to 3 for each (e.g., 3 = fully and correctly explained the four ground rules, 2 = partially explained the four ground rules, 1 = did not explain the four ground rules). Two-way mixed effects Intraclass Correlation Coefficient (ICC) analysis testing for absolute agreement between coders for the interviewer behaviors across the sample of 30 interviews revealed good inter-rater reliability for each of the interviewer behaviors, (i) six phases, ICC = 0.937 (95% CI 0.867; 0.970), (ii) correct ground rules, ICC = 1.000, (95% CI 1.00; 1.00), (iii) use of ground rules across three phases, ICC = 0.944 (95% CI 0.889; 0.972), (iv) four TED questions, ICC = 0.865, (95% CI 0.498; 0.964), and (v) rapport building, ICC = 0.757 (95% CI 0.096; 0.935). Mean scores for each behavior as a function of interviewer revealed a very high level of adherence to the protocol for each behavior, with no significant differences across interviewers for each behavior, all *F*s < 1.120, all *p*s > 0.299 (see Table 3).

**Table 3:** Mean interviewer protocol adherence scores across interviewer 1 and 2 (dip sample of 30 interviews).

	Mean (SD) 95% CI	
	Interviewer 1	Interviewer 2
Behavior		
Six phases	2.74 (0.46) 2.48, 2.99	2.74 (0.46) 2.48, 2.99
Ground rules	2.80 (0.41) 2.57, 3.03	2.87 (0.35) 2.67, 3.06
Apply ground rules correctly	2.80 (0.41) 2.57, 3.02	2.86 (0.35) 2.67, 3.05
TED questions	2.73 (0.46) 2.48, 2.99	2.73 (0.46) 2.48, 2.99
Verbal rapport	2.93 (0.26) 2.79, 3.08	2.80 (0.41) 2.57, 3.03

## 3. Results

### 3.1. Analysis approach

A series of 3 (Language: South Asian L1; South Asian L2; British L1) X 2 (Veracity: Truth; Lie) ANOVAs were conducted across the three dependent variables (Event details; Verifiable sources; Complications), applying Bonferroni’s correction as appropriate. Main effects are reported in the results text, interactions are displayed in Table 4.

**Table 4:** Event details, complications, verifiable sources, and plausibility interactions.

	Mean (SD) 95% CI		
	South Asian L1 (Hindi)	British L1	South Asian L2 (English)
Event details			
Liar	23.68 (5.86) 20.62, 26.75	26.76 (5.37) 23.89, 29.64	22.78 (4.00) 19.29, 26.28
Truthteller	71.39 (9.80) 68.39, 74.39	63.00 (7.18) 60.00, 66.00	66.45 (12.09) 62.71, 70.19
Complications			
Liar	3.09 (1.23) 2.51, 3.67	2.00 (1.35) 1.43, 2.57	1.22 (1.09) 0.65, 1.79
Truthteller	5.39 (1.44) 4.82, 5.97	6.30 (1.58) 5.71, 6.90	6.05 (1.70) 5.44, 6.66
Verifiable sources			
Liar	2.32 (1.21) 1.69, 2.95	1.56 (1.08) 0.94, 2.18	4.09 (1.91) 3.44, 4.74
Truthteller	5.91 (2.09) 5.29, 6.53	5.87 (1.39) 5.22, 6.52	6.50 (1.82) 5.80, 6.52
Plausibility			
Liar	3.82 (1.37) 3.33, 4.31	2.92 (0.99) 2.51, 3.32	3.09 (0.99) 2.66, 3.52
Truthteller	4.35 (1.27) 3.87, 4.83	5.22 (0.95) 4.79, 5.65	3.90 (1.20) 3.44, 4.36

#### 3.1.1. Event details

There was a significant main effect of veracity for event details, *F*(1, 130) = 1022.73, *p* < 0.001, *η*_p_^2^ = 0.89. All liars provided fewer event details than truthtellers (*M*_Liar_ = 24.49, SD = 5.35, 95% CI 22.58, 26.21; *M*_Truth_ = 66.97, SD = 10.26, 95% CI 65.09, 68.84, *d* = 5.44). The main effect of language was non-significant, *F*(1, 130) = 1.96, *p* = 0.146, (*M*_SA L1_ = 47.54, SD = 25.42, 95% CI 45.25, 49.82; *M*_SA L2_ = 44.62, SD = 23.66, 95% CI 42.28, 46.96; *M*_British_ = 44.13, SD = 19.32, 95% CI 42.69, 47.09). The language X veracity interaction was significant, *F*(2, 130) = 6.59, *p* = 0.002, *η*_p_^2^ = 0.91. South Asian truthtellers provided significantly more event details than South Asian liars in both first (L1), and second (L2) languages, all *p*s < 0.001, languages (see Table 4).

#### 3.1.2. Complications

There was a significant main effect of veracity for complications, *F*(1, 130) = 248.84, *p* < 0.001, *η*_p_^2^ = 0.66. All liars provided fewer complications than truthtellers (*M*_Liar_ = 2.10, SD = 1.43, 95% CI 1.77, 2.44; *M*_Truth_ = 5.92, SD = 1.56, 95% CI 5.57, 6.26, *d* = 1.06). The main effect of language was non-significant, *F*(2, 130) = 2.38, *p* = 0.096 (*M*_SA L1_ = 4.24, SD = 1.23, 95% CI 3.83, 4.66; *M*_SA L2_ = 3.63, SD = 1.09, 95% CI 3.21 4.06; *M*_British_ = 4.15, SD = 1.35, 95% CI 3.75, 4.55). The language X veracity interaction was significant, *F*(2, 130) = 10.05, *p* < 0.001, *η*_p_^2^ = 0.13. South Asian truthtellers provided significantly more complications than South Asian liars in both first (L1) and second (L2) languages all *p*s < 0.001 (see Table 4).

#### 3.1.3. Verifiable sources

There were significant main effects of veracity, *F*(1, 130) = 152.99, *p* < 0.001, *η*_p_^2^ = 0.54 and language *F*(2, 130) = 11.44, *p* < 0.001, *η*_p_^2^ = 0.15, for verifiable sources. Liars provided fewer verifiable sources (*M*_Liar_ = 2.66, SD = 1.19, 95% CI, 2.72, 3.04) than truthtellers (*M*_Truth_ = 6.09, SD = 1.78, 95% CI, 5.70, 6.49, *p* = <0.001, *d* = 0.82). South Asian L1 (*M*_SA L1_ = 4.12, SD = 2.49, 95% CI, 3.67, 4.55) participants provided fewer verifiable details than South Asian L2 (*M*_SA L2_ = 5.29, SD = 2.21, 95% CI, 4.80, 7.78), *p* = 0.003, *d* = 0.50. The language X veracity interaction was significant, *F*(2, 130) = 3.93, *p* = 0.022, *η*_p_^2^ = 0.06 (see Table 4). South Asian truthtellers provided significantly more verifiable details than South Asian liars in both first (L1) and second (L2) languages (see Table 4), all *p*s < 0.001. South Asian (L1) liars provided fewer verifiable details than South Asian (L2) liars, *p* < 0.001.

#### 3.1.4. Plausibility

There was a significant main effect of veracity for plausibility ratings, *F*(1, 130) = 38.59, *p* < 0.001, *η*_p_^2^ = 0.23, All liars were rated less plausible (*M*_Liar_ = 3.27, SD = 1.18, 95% CI, 3.01, 3.54) than truthtellers, (*M*_Truth_ = 4.52, SD = 1.26, 95% CI, 4.21, 4.77, *d* = 0.20). The main effect of language was non-significant, *F*(2, 130) = 3.85, *p* = 0.024 (*M*_SA L1_ = 4.09, SD = 1.33; *M*_SA L2_ = 3.47, SD = 1.16; *M*_British_ = 4.02, SD = 1.51). The veracity X language interaction was significant, *F*(2, 130) = 8.138, *p* < 0.001, *η*_p_^2^ = 0.11. British (English speaking) truthtellers were rated more plausible than South Asian L1 and L2 truthtellers, all *p*s < 0.032 (Bonferroni adjusted). British (English speaking) liars were rated as less plausible than South Asian (L1 Hindi) liars, *p* = 0.023 (Bonferroni adjusted).

#### 3.1.5. Post interview questionnaire

##### 3.1.5.1. Motivation

Overall, self-reported motivation to comply with researcher instructions was high, *M* = 6.13 (SD = 0.87). Main effects of veracity (*M*_Liar_ = 6.16, SD = 0.91; *M*_Liar_ = 6.11, SD = 0.83) and culture (*M*_British_ = 6.27, SD = 0.89; *M*_SA_ = 6.06, SD = 0.85) were non-significant, all *F*s < 1.671, all *p*s > 0.194. However, the veracity X culture interaction was significant with British liars self-reported more motivation (*M*_British_ = 6.60, SD = 0.76) than South Asian liars (*M*_SA_ = 5.91, SD = 0.90), *p* = 0.001. All other interactions were non-significant, *p* = 0.173.

##### 3.1.5.2. Adherence

Overall, self-reported adherence to researcher instructions (as a function of condition) was high, *M* = 6.32 (SD = 0.72). Main effects of veracity (truthteller, liar) and culture (British, South Asian) were non-significant, as was the veracity X culture interaction, all *F*s < 0.001, all *p*s > 0.269.

##### 3.1.5.3. Difficulty

Overall, participants self-reported the interview to be neither easy nor difficult (*M* = 4.34, SD = 0.50). Main effects of veracity, *F*(1, 132) = 195.167, *p* < 0.001, *η*_p_^2^ = 0.60, and culture (British, South Asian), *F*(1, 132) = 18.463, *p* < 0.001, *η*_p_^2^ = 0.12, were significant. All liars found the interview more difficult (*M*_Liar_ = 3.04, SD = 1.20), than truthtellers, (*M*_Truth_ = 5.71, SD = 1.26), *p* < 0.001. South Asian participants found the interview more difficult than British participants (*M*_SA_ = 4.05, SD = 1.68; *M*_British_ = 4.88, SD = 1.94). The veracity X culture interaction, was significant, *F*(1, 132) = 7.787, *p* = 0.006, *η*_p_^2^ = 0.56. South Asian truthtellers and liars found the interview more difficult than British truthtellers (*M*_SA Truth_ = 5.21, SD = 1.23; *M*_British Truth_ = 6.65, SD = 0.65) and liars (*M*_SA Liar_ = 2.93, SD = 1.25; *M*_British Liar_ = 3.43, SD = 0.95), all *p*s < 0.002.

##### 3.1.5.4. Understanding

Overall, participants self-reported understanding of the interviewer’s questions was high (*M* = 6.78, SD = 0.50). Main effects of veracity (truthteller, liar) and culture (British, South Asian) were non-significant, as was the veracity X culture interaction, all *F*s < 4.206, all *p*s > 0.042.

## 4. Discussion

There is a paucity of research concerned with verbal veracity cues in forensic interview contexts with bilinguals from non-western cultures. We investigated the occurrence of several verbal behaviors that have emerged from North American and Western European monolingual research as promising cues to veracity. To investigate differences and similarities in verbal behaviors between cultures as a function of veracity and interview language (L1 and L2), South Asian participants were interviewed in first and second languages, whereas British participants were interviewed in English only.

Irrespective of interview language (L1; L2) or culture (South Asian; British), all liars verbalized significantly fewer event details, verifiable information, and complications than truthtellers. This pattern of results is consistent with our expectations and with previous research (e.g., DePaulo et al., 2003; Vrij, 2008; Leal et al., 2018; Vrij and Vrij, 2020; Vrij et al., 2021b, 2022) and advances understanding by suggesting these verbal behaviors are stable across cultures (British and South Asian) for liars and truthtellers, including when interviews are conducted in a second language. This latter finding is arguably the most intriguing, given the often-made assumption that speaking in a second language degrades the quality of discourse (Taylor et al., 2014) since speaking in a second language places additional demands on neural processing (Perani and Abutalebi, 2005) which makes conversations more challenging (Ullman, 2001; Da Silva and Leach, 2013). Nonetheless, as predicted by cognitive load theories, the increased cognitive demand typically associated with being deceptive has impacted verbal behavior similarly across cultures, irrespective of interview language, as has been reported by others.

We expected that second language speakers would include less of some of the verbal cues than the first language speakers due to the additive effect of cognitive load stemming from language and veracity. Our results do not support this hypothesis since main effects revealed that South Asian participants in the L2 condition provided more verifiable sources than their L1 language South Asian counterparts. Furthermore, South Asian L2 liars again provided more verifiable sources than their L1 counterparts. That said, the additional cognitive loading imposed by speaking in a second language is neither consistent nor static. L2 practice can lighten cognitive load whereby second language conversations become ‘easier’ because as proficiency improves control mechanisms strengthen, significantly reducing multiple language interference (e.g., Costa et al., 2006; Albl-Mikasa et al., 2020; Liu et al., 2021). Therefore, it seems sensible to expect that bilingual L2 proficiency may moderate cross-cultural differences in verbal veracity cues in an interview context (e.g., Evans et al., 2013).

Here, our bilingual participants were studying at a British university, and all indicated regular, daily use of L2. Indeed, responses to the language questionnaire indicate many participants preferred to speak in English rather than Hindi while at university or had no preference, and so participants were clearly comfortable speaking either language. Accordingly, it is possible that our findings are limited to those with a high level of English language proficiency. Objective language proficiency evaluations that map directly onto cognitive load may be important for understanding possible additive effects for fully understanding the utility of verbal cues. Furthermore, since second language ability develops variously according to exposure to relevant language-learning and cultural contexts, if exposure is limited and/or intermittent, second language ability may be inadequate, despite initial appearances (see Francis, 2006).

Our results are broadly consistent with prior literature, and reinforce an observation made elsewhere (Taylor et al., 2017), which is that cultural variations in interpersonal norms and memory encoding may manifest as ‘main effect’ differences in the behaviors observed from two cultures. This does not affect the evidence for aggregate effects of veracity across our dependent variables. But it does impact any effort to give a point estimate (Nahari et al., 2019) that answers practical questions centered on how to differentiate whether a person of interest is lying. The amount of information that would provide the best cut off between liar and truth-teller may be different for each culture.

Despite the inconsistencies of prior research, we expected that liars across all conditions would judged less plausible that truthtellers. Our results support this hypothesis whereby all liars were rated less plausible. We found no differences in plausibility ratings as a function of L1 or L2 for South Asian participants, but British (English speaking) truthtellers were rated more plausible than South Asian L1 and L2 truthtellers. Further, British (English speaking) liars were rated as less plausible than South Asian liars interviewed in L1 (Hindi). These results suggest that some judges may tend to use more extreme ratings when judging British speakers, which may reflect the cultural background of our raters who were all monolingual British. However, these findings are braodly in line with research suggesting an a more pronounced observer lie bias when judging non-native speakers (Da Silva and Leach, 2013; Evans and Michael, 2014; Wylie et al., 2022), although this may not be the case were judges and coders are bilingual and culturally matched to the interviewee, since the assessments of plausibility are likely to vary depending on the knowledge and expertise of those making a judgment. This would speak to questions concerning whether cross cultural interviews should be conducted in a second language or via an interpreter, perhaps.

That some cues manifested differently across our two cultural groups raises a challenge for research and practice in forensic interview contexts moving forward. As Taylor and colleagues summarize (Taylor et al., 2017), the challenge this poses for the research community is that research could become reductionary, with researchers introducing “new moderators and cut their samples into smaller ‘cultures’” (Hope et al., 2022). This reinforces the view that research moving forward should concern itself less with providing ways to determine veracity and focus on techniques that improve the interaction between interview techniques, interviewer, and the person of interest being interviewed. A constructive interaction is likely to provide the best opportunity to derive checkable evidence that aids an investigation (see also Dando and Ormerod, 2020) rather than relying on research to project an absolute (but arbitrary) value of number of cues related to truthtellers and liars. Cultural differences in cue generation found in this research suggests that monolingual British interviewers and observers may well misjudge the veracity of British and South Asian liars and truthtellers, irrespective of whether they are basing their judgments on plausibility, numbers of complications, or verifiable sources.

Whilst our findings suggest that verifiability, and plausibility may be useful cues to deception, and that generally speaking they appear robust across cultures, how they manifest in absolute terms will vary. It will be interesting to determine if this remains true for cues that are not about information but about other elements of the interaction, such as relational humor (Hamlin et al., 2020) and rapport (Gabbert et al., 2021; Dando C. et al., 2022). We might hypothesize, for example, that if a second language person of interest might focus entirely on providing information, the wider facets of interaction suffer, and this may also expose their deception.

The limitations of our research are clear and ubiquitous. The paradigm employed allowed us to control several variables toward unpicking differences and similarities in verbal behaviors across cultures, but our approach may reduce generalizability. We culturally matched interviewer and interviewee, which maps onto the paradigms employed by some researchers (e.g., Cheng and Broadhurst, 2005; Leal et al., 2018), but differs from other approaches (e.g., Elliott and Leach, 2016; Akehurst et al., 2018). Our interviewers were kept constant, whereby we kept the same bilingual interviewer for the bilingual group and a second monolingual interviewer for the monolingual group. This reduces potentially confounding interviewer behavior variables, but conversely introduces the possibility that our results are confounded by behaviors specific to each interviewer. That said, we used an interview protocol, and the single/multiple interviewer tension is common to all experimental interviewing research such as this.

We used transcripts only as the basis for plausibility judgments, which others have found to leverage higher discrimination accuracy for second language interviews than when visual + audio and/or audio only excepts are utilized (Akehurst et al., 2018). We sought to optimize accurate judgments by eliminating the non-verbal behavior which can decrease accuracy (Vrij et al., 2010; Bull et al., 2019; Sandham et al., 2020; Dando C. J. et al., 2022). However, in doing so we have reduced a complex social interaction to a series of sentences, thus reducing a multifaceted social interaction. It is likely that the value of verbal behavior is far more. Hence our findings may be most relevant for transcript only judgments. Further research centered on the utility of verbal cues when listening to the audio versus listening to the audio plus observing the social interaction would add to our results.

Translation of the Hindi interviews into English has been highlighted by others as a limitation, since around 10% of information may be lost in the process of translation (see Ewens et al., 2017; Leal et al., 2018). This may have impacted our results, although information loss is likely small and translation is a limitation for all bilingual research, irrespective of discipline. We only coded verbalized information within each of the three categories once. Hence, there were no within category duplications (i.e., event details; complications; verifiable sources). However, it is possible that some information items were not mutually exclusive, since an item of event information may also map onto our definition of a verifiable source, for example. This possibility was controlled for by analyzing each category individually, which maps onto the approach employed by others and does not negate our findings. Finally, South Asian liars self-reported being slightly less motivated to be deceptive than British liars. The locus of this result is unclear and the literature in this regard is sparce. It maybe that motivation was influenced by an interplay of intercultural communication, cultural group membership and social moral values (see Giles et al., 2019).

Finally, *a-priori* power analysis (Faul et al., 2007) revealed our sample size was adequate to detect large effects, but not powerful enough to detect small effects and so future research might consider larger sample sizes toward a more nuanced understanding, although the impact of small effect sizes for applied research is currently the subject of discussion (see Götz et al., 2022; Primbs et al., 2022).

Despite the limitations of research of this nature, our findings offer novel insights into the impact of two contextual variables, culture and language on verbal behavior in face-to-face forensic interviews which were information gathering in nature and designed to amplify potential verbal veracity cues. Our results are promising in terms of again highlighting that while context is an important consideration, irrespective of culture and interview language context, impoverished, simple verbal accounts should trigger a ‘red flag’ for further attention.

## Data availability statement

The datasets presented in this study can be found in online repositories. The names of the repository/repositories and accession number(s) can be found below: https://osf.io/p72ur/?view_only=aa29c3c9cd4f463996cb08e85ca3f473.

## Ethics statement

The studies involving human participants were reviewed and approved by the Lancaster University Ethics Committee. The patients/participants provided their written informed consent to participate in this study.

## Author contributions

CD and PT designed the paradigm. AS, CD, and PT steered the research question and data analysis and wrote the introduction, method, results, and discussion. All authors contributed to the article and approved the submitted version.

## Conflict of interest

The authors declare that the research was conducted in the absence of any commercial or financial relationships that could be construed as a potential conflict of interest.

## Publisher’s note

All claims expressed in this article are solely those of the authors and do not necessarily represent those of their affiliated organizations, or those of the publisher, the editors and the reviewers. Any product that may be evaluated in this article, or claim that may be made by its manufacturer, is not guaranteed or endorsed by the publisher.
